# How to Apply the Soda Remedy in Burns and Scalds

**Published:** 1883-03

**Authors:** 


### HOW TO APPLY THE SODA REMEDY IN BURNS AND SCALDS.

It is now many years ago that the author, while engaged in some investigations as to the qualities and effects of the alkalies in inflammations of the skin, etc., was fortunate enough to discover that a saline lotion or saturated solution of the bicarbonated soda in either plain water or camphorated water, if applied speedily or as soon as possible, to a burned or scalded part was most effectual in immediately relieving the acute burning pain, and when the burn was only superficial or not severe, removing all pain in the course of a very short time, having also the very great advantage of cleanliness and, if applied at once, of preventing the usual consequences —a painful blistering of the skin, separation of the epidermis and, perhaps, more or less of suppuration.

For this purpose all that is necessary is to cut a piece of lint or old soft rag or even thick blotting paper, of a size sufficient to cover the burned or scalded parts and to keep it constantly well wet with the sodaic lotion so as to prevent its drying. By this means it usually happens that all pain ceases in from a quarter to half an hour, or even in much less time. When the main part of a limb, such as the hand and forearm or the foot and leg, has been burned it is best when practicable to plunge the part at once into a jug or pail or other convenient vessel filled with the soda lotion, and keep it there until the pain subsides, or the limb may be swathed or encircled with a surgeon's cotton bandage previously soaked in the saturated solution and kept constantly wet with it, the relief being usually immediate provided the solution be saturated and cold. What is now usually sold as bicarbonate of soda is what I have commonly used and recommended, although this is well known to vary much in quality according to where it is manufactured, but it will be found to answer the purpose, although probably Howard's is most to be depended on, the common carbonate being too caustic. It is believed that a large proportion of medical practitioners are still unaware of the remarkable qualities of this easily applied remedy, which recommends itself for obvious reasons.—*F. Peppercorne in Popular Science Monthly for March.*

---

CHILDREN POISONED WITH TOBACCO.—In one of the schools of Brooklyn a boy thirteen years old, naturally very quick and bright, was found to be growing dull and fitful. His face was pale, and he had nervous twitchings. He was obliged to quit school. Inquiry showed that he had become a confirmed smoker of cigarettes. When asked why he did not give it up, he shed tears, and said that he had often tried but could not. The growth of this habit is insidious, and its effects ruinous. The eyes, the brain, the nervous system, the memory, the power of application, are all impaired by it, "It 's nothing but a cigarette" is, really, "It 's nothing but poison." German and French physicians have recently protested against it, and a convention of Sunday and secular teachers was recently held in England to check it. It was presided over by an eminent surgeon of a Royal Eye Infirmary, who stated that many of the diseases of the eye were directly caused by it. Parents, save your children from this vice, if possible! Do not allow them to deceive you. In future years they will rise up and bless you for restraining them.

GOVERNOR SHELDEN, of New Mexico, in a recent interview, said he did not see any immediate prospect of the Territory becoming a State. A State Government would largely increase taxation, while the territorial form has not yet been sufficiently systematized or advanced to give assurance that a State Government would be such as comports with the modern idea. Touching the condition and material prospects of the Territory, the Governor stated that there were at least ten million acres offered, adapted to all kinds of vegetables, fruits and cereals, and sufficient crops could be raised to supply one million people. As a country for stock raising there is none that is superior. New Mexico has an abundance—gold, silver, copper, lead and iron, and considerable wealth undeveloped in timber and coal. Admitting that at this time the Territory is not so prosperous as it should be, he says the reason is that the general production is not so large as it should be, which necessitates too large purchases from abroad to feed and clothe the people.

